# Effects of three aerobic exercise modalities (walking, running, and cycling) on circulating brain-derived neurotrophic factor in older adults: a systematic review and meta-analysis

**DOI:** 10.3389/fnagi.2025.1673786

**Published:** 2025-09-25

**Authors:** Yang Cheng, Yuxiang Liu, Jing Ma, Zhen Li, Enli Han, Shumin Bo

**Affiliations:** ^1^School of Kinesiology and Health, Capital University of Physical Education and Sports, Beijing, China; ^2^College of Physical Education and Sport, Langfang Normal University, Langfang, Hebei, China; ^3^School of Physical Education and Sport Science, Fujian Normal University, Fuzhou, Fujian, China

**Keywords:** aerobic exercise, brain-derived neurotrophic factor, older adults, meta-analysis, walking, running, cycling

## Abstract

**Background:**

Recent studies indicate that older adults (aged 55 years and above) represent a critical period for changes in circulating brain derived neurotrophic factor (BDNF) levels and cognitive function. Regular aerobic exercise (AE) has been recognized as a promising non-pharmacological strategy to influence neuroplasticity and cognitive function, primarily through the regulation of BDNF. However, inconsistent findings have been reported regarding the specific effects of three AE modalities—walking, running, and cycling—on circulating BDNF levels in older adults. This study aimed to systematically evaluate the effects of these AE modalities on BDNF levels in the elderly through a meta-analysis, and to further compare the relative effectiveness of various exercise protocols using network meta-analysis.

**Methods:**

A systematic search was conducted from database inception to June 10, 2025, in PubMed, Web of Science, Embase, the Cochrane Library, and Scopus. The methodological quality and risk of bias of the included studies were assessed using the Cochrane Risk of Bias 2 tool. Meta-analysis and network meta-analysis were conducted using Stata software (version 18, StataCorp LLC, United States).

**Results:**

A total of 17 studies involving 900 older participants were included. Meta-analysis indicated that three AE modalities significantly increased circulating BDNF levels (SMD = 0.62, 95% CI: 0.06 to 1.18, *p* = 0.03). Subgroup analysis revealed that the intervention effect was significantly influenced by participants’ health status (*p* < 0.01). Specifically, the interventions had positive effects in healthy individuals and those with mild cognitive impairment. Exercise-related variables such as modality, intensity, and the interval were identified as potential moderators. Network meta-analysis demonstrated that protocols involving low-intensity short-duration walking (WLS) were superior to other exercise protocols (*p* < 0.05), and protocols involving moderate-intensity short-duration walking (WMS) were more effective in increasing BDNF levels than high-intensity long-duration walking (*p* < 0.05). Surface under the cumulative ranking curve results further supported the superiority of WLS (99.9%) and WMS (83.7%) over other exercise protocols.

**Conclusion:**

Walking, running, and cycling are effective for improving circulating BDNF levels in older adults; however, the magnitude of improvement depends on participants’ health status and specific exercise prescription. Interventions involving walking at low to moderate intensity demonstrated favorable efficacy. This effect may be more favorable in healthy individuals and those with mild cognitive impairment. Future studies should further investigate the influence of total exercise volume on outcomes and adopt more rigorous and standardized protocols to facilitate the development of standardized exercise strategies, thereby improving comparability and reducing heterogeneity in future analyses.

**Systematic review registration:**

https://www.crd.york.ac.uk/PROSPERO/, Identifier CRD420251068909.

## 1 Introduction

With advancing age, the progressive accumulation of deleterious cellular components and molecular markers significantly impairs the body’s ability to cope with internal and external stressors, rendering the brain increasingly susceptible to a state of chronic, low-grade neuroinflammation (Rezaee et al., 2022). As one of the core systems involved in responding to and resisting stress, the nervous system exhibits high sensitivity to such exposure. Consequently, any risk factors associated with the nervous system, including psychological stress and emotional trauma, may induce neuronal apoptosis and synaptic plasticity impairments (Barde, 2025). Notably, aging further intensifies these processes, leading to brain atrophy, microstructural white matter damage, and alterations in neural activity patterns (Raz et al., 2010), thereby elevating the risk of psychiatric and neurodegenerative disorders in the elderly, including mild cognitive impairment (MCI), Alzheimer’s disease (AD), and Parkinson’s disease (PD) (Mattson and Magnus, 2006). Although cognitive decline often occurs with aging (manifested as a high incidence of cognitive disorders among older adults) (Morrison and Baxter, 2012), existing evidence has demonstrated that certain health-promoting behaviors may enhance cognitive performance and delay its deterioration. Among these, physical exercise has been identified as one of the most promising interventions due to its cost-effectiveness, accessibility, and efficiency (Erickson et al., 2022; Gates et al., 2013).

While general exercise guidelines are available for all ages, whether these guidelines are optimal or sufficient for improving cognitive function in older adults remains debated, and the underlying mechanisms are not fully understood. Therefore, further investigation into the cognitive benefits of physical exercise in aging populations remains critically important. A systematic review revealed that supervised exercise interventions significantly improved cognitive function in adults aged 50 years and older, regardless of whether participants were cognitively intact or exhibited cognitive impairments (Northey et al., 2018). Similarly, recent findings have indicated that moderate-intensity aerobic exercise (AE) not only contributes to improved glycemic control in older individuals with type 2 diabetes (T2D), but also enhances cerebral vascular hemodynamics, and significantly increases circulating levels of brain-derived neurotrophic factor (BDNF) (Ploydang et al., 2023). These findings suggest that the cognitive benefits of exercise may be mediated through multiple neurobiological mechanisms, among which BDNF has been identified as a critical regulatory factor underlying exercise-induced improvements in cognitive function.

BDNF, a frequently studied dimeric polypeptide and member of the neurotrophin family, plays a crucial role in neuronal differentiation, axonal growth, survival, and synaptic plasticity. However, its levels naturally decline with age (von Bohlen und Halbach, 2010). BDNF is widely expressed in the brain, particularly in the hippocampus, amygdala, cerebral cortex, and cerebellum, and it can potentially cross the blood–brain barrier to play a key role in enhancing learning, memory, and cognitive function (Jemni et al., 2023). Notably, the transport of BDNF across the blood–brain barrier may be influenced by its form. For instance, platelet-bound BDNF may be released more slowly than freely circulating BDNF, potentially altering its bioavailability and affecting peripheral BDNF levels (Dell'Oste et al., 2024). BDNF is initially synthesized in its precursor form (proBDNF), which is then cleaved by proteases into its biologically active mature form (mBDNF) (Antonijevic et al., 2024). mBDNF is released during synaptic activity or neuronal excitation (including physical exercise), where it binds to its high-affinity receptor TrkB (tropomyosin receptor kinase B), activating a series of downstream signaling pathways which may be associated with potential benefits for cognitive function and synaptic plasticity (Li et al., 2022).

In addition to its effects on neuroplasticity, BDNF has been associated with increased hippocampal volume in older adults. Studies have demonstrated that AE can significantly improve circulating BDNF levels, increases hippocampal volume, and enhances the gray matter volume in several cortical regions (Firth et al., 2018; Prakash et al., 2010; Sharma et al., 2024). For instance, Erickson et al. (2011) conducted a one-year moderate-intensity walking training program (50 to 75% HRR) in healthy older adults and found significant increases in hippocampal volume, which were closely associated with elevated serum BDNF levels. However, inconsistent findings were reported by Enette et al. (2020), who conducted a 9-week cycling training program for elderly individuals with AD. Despite significant improvements in participants’ 6-min walking test performance and quality of life, no significant changes in plasma BDNF levels or cognitive function after the intervention. Similarly, Maass et al. (2016) reported that a 3-month AE intervention did not significantly alter circulating BDNF levels or cognitive performance in the elderly.

Despite some inconsistencies, clinical trial evidence suggests that appropriately designed AE protocols can significantly modulate BDNF levels, enhance brain structure, and contribute to the prevention of age-related psychiatric and neurodegenerative disorders (Rezaee et al., 2022). Notably, different exercise types exert differential effects on BDNF regulation. In particular, compared to resistance exercise, AE appears to confer greater benefits in promoting neuroplasticity and enhancing cognitive function (Hvid et al., 2017; Tsai et al., 2019). However, the current literature remains inconclusive regarding the specific effects of three AE modalities—walking, running, and cycling—on circulating BDNF levels in older adults. This study aims to systematically evaluate the impact of these AE modalities on circulating BDNF in the elderly. Furthermore, a network meta-analysis will be conducted to identify the most effective AE protocols. Restricting the analysis to these modalities enables a more coherent comparison of their effects and enhances the applicability of findings to practical exercise prescriptions. This study is expected to clarify the comparative efficacy of commonly practiced AE protocols in modulating BDNF and provide a theoretical basis for developing individualized exercise prescriptions that may support cognitive health in aging populations, while also offering methodological references for the prevention of cognitive decline–related psychiatric and neurodegenerative conditions.

## 2 Methods

This meta-analysis has been registered on the International Prospective Register of Systematic Reviews (PROSPERO), with registration number CRD420251068909, and was conducted in accordance with systematic reviews and meta-analyses, as outlined by the PRISMA statement (Page et al., 2021).

### 2.1 Search strategy

A systematic search was conducted from database inception to June 10, 2025, in PubMed, Web of Science, Embase, the Cochrane Library, and Scopus. Boolean operators (AND, OR) were used to search randomized controlled trials (RCTs) related to the effects of walking, running, and cycling on BDNF levels in older adults. The search strategy incorporated the following terms: “Exercise,” “Physical Exercise,” “Aerobic Training,” “Aerobic Exercise,” “Walking,” “Jogging,” “Running,” “Cycling,” “Bicycling,” “Aged,” “Elder,” “Older Adults,” “Older Adult,” “Elderly,” “Brain-Derived Neurotrophic Factor,” and “BDNF.” A detailed search strategy for PubMed is provided in Table 1.

**Table 1:** Detailed search strategy using PubMed as an example.

Query	Query search terms
#1	((((((((((((((((((((((“Exercise”[Mesh]) OR (Exercises[Title/Abstract])) OR (Exercise, Physical[Title/Abstract])) OR (Exercises, Physical[Title/Abstract])) OR (Physical Exercise[Title/Abstract])) OR (Physical Exercises[Title/Abstract])) OR (Exercise, Aerobic[Title/Abstract])) OR (Aerobic Exercise[Title/Abstract])) OR (Aerobic Exercises[Title/Abstract])) OR (Aerobic training[Title/Abstract])) OR (Exercises, Aerobic[Title/Abstract])) OR (Exercise Training[Title/Abstract])) OR (Exercise Trainings[Title/Abstract])) OR (Training, Exercise[Title/Abstract])) OR (Trainings, Exercise[Title/Abstract])) OR (Physical Activity[Title/Abstract])) OR (Activities, Physical[Title/Abstract])) OR (Activity, Physical[Title/Abstract])) OR (Physical Activities[Title/Abstract])) OR (Exercise[Title/Abstract])) OR (((“Running”[Mesh]) OR (running[Title/Abstract])) OR (Jogging[Title/Abstract]))) OR (((“Walking”[Mesh]) OR (Nordic Walking[Title/Abstract])) OR (Walking[Title/Abstract]))) OR (((“Bicycling”[Mesh]) OR (Cycling[Title/Abstract])) OR (Bicycling[Title/Abstract]))
#2	(((((((((“Aged”[Mesh]) OR (Aged[Title/Abstract])) OR (Elders[Title/Abstract])) OR (Elder[Title/Abstract])) OR (Older Adults[Title/Abstract])) OR (Adult, Older[Title/Abstract])) OR (Adults, Older[Title/Abstract])) OR (Older Adult[Title/Abstract])) OR (Elderly[Title/Abstract]))
#3	(((((“Brain-Derived Neurotrophic Factor”[Mesh]) OR (Brain-Derived Neurotrophic Factor[Title/Abstract])) OR (Brain Derived Neurotrophic Factor[Title/Abstract])) OR (Factor, Brain-Derived Neurotrophic[Title/Abstract])) OR (Neurotrophic Factor, Brain-Derived[Title/Abstract])) OR (BDNF[Title/Abstract])
#4	#1 AND #2 AND #3

### 2.2 Eligibility criteria

#### 2.2.1 Inclusion criteria

1. Study Population: Older adults aged ≥55 years, regardless of sex or health status; this age cutoff was chosen since recent studies indicate it is a critical period for changes in BDNF levels and cognitive function (Kim et al., 2025; Wang et al., 2024).
2. Intervention: Participants perform AE in the modality of walking, running, or cycling, with exercise interventions lasting a minimum of 4 weeks (Cardoso et al., 2024; Gholami et al., 2025; Ribeiro et al., 2021), if an intervention simultaneously involved two of these modalities, it was also eligible to comprehensively capture the effects of exercise on BDNF.
3. Control Group: The control group consisted of participants engaging in non-AE (e.g., stretching) or maintaining their usual lifestyle.
4. Outcome Measures: Studies have reported plasma or serum BDNF levels before and after the intervention, with no restrictions on detection methods.
5. Study Design: RCTs.

#### 2.2.2 Exclusion criteria

Studies were excluded if they were non-randomized controlled trials, self-controlled trials, animal studies, duplicate publications, review articles, or conference abstracts. Studies with unclearly described interventions, or those involving exercise modalities other than walking, running, or cycling (e.g., combined aerobic and resistance training, Tai Chi, dance), were also excluded. These three modalities were selected to reduce heterogeneity across exercise types and to facilitate the comparison of specific intervention protocols in the network meta-analysis. In addition, studies that did not report changes in BDNF levels, lacked extractable statistical data, or included control groups involving resistance training or other interventions that could confound comparisons were excluded.

### 2.3 Study selection and data extraction

After removing duplicate references using EndNote (version 20.6, Clarivate, United States), two authors (Y.C. and Y.L.) independently screened article titles and abstracts according to the pre-established inclusion and exclusion criteria. Subsequently, potentially eligible studies were selected for full-text review to determine final eligibility. The two authors extracted study characteristics using Microsoft Excel, including first author, publication year, participant demographics, specific parameters of the exercise intervention (e.g., type, frequency, intensity, duration), and outcome measures. Any disagreements were resolved through discussion, and if necessary, consultation with a third author (Z.L.) to ensure accuracy and consistency.

### 2.4 Bias risk and methodological quality assessment of included studies

The Cochrane Risk of Bias 2 (RoB 2) tool was used to assess the risk of bias and methodological quality of the included studies (Sterne et al., 2019). This tool consists of five domains: (1) randomization process; (2) deviations from intended interventions; (3) missing outcome data; (4) measurement of the outcome; and (5) selection of the reported result. Based on a comprehensive assessment of these five domains, the two authors (Y.C. and Y.L.) independently evaluated the studies using three categories: “low risk,” “some concerns,” or “high risk.” Any discrepancies in the assessment were resolved through discussion.

### 2.5 Statistical methods

Meta-analysis was performed using Stata software (version 18, StataCorp LLC, United States) (Antoniou et al., 2019). Given the inconsistency in measurement units across studies, the standardized mean difference (SMD) and its 95% confidence interval (95% CI) were used for the pooled effect analysis, with Hedges’ g applied to correct for biases arising from small sample sizes (Taylor and Alanazi, 2023). Heterogeneity was assessed using the I^2^ statistic, where values of I^2^ at 25, 50, and 75% indicate low, moderate, and high levels of heterogeneity, respectively. If I^2^ exceeded 50%, indicating significant heterogeneity, a random-effects model was used to pool the effect sizes; otherwise, a fixed-effects model was applied (Higgins et al., 2003). The interpretation of effect size was as follows: SMD < 0.4 indicates a small effect, 0.4–0.8 indicates a medium effect, and SMD > 0.8 indicates a large effect (Kons et al., 2023). To explore the potential sources of heterogeneity, subgroup analysis was performed for categorical variables, while meta-regression was used for continuous variables. Funnel plots were generated, and publication bias was quantitatively assessed using Egger’s test. If significant bias was detected, results were further corrected using the trim-and-fill method. Finally, sensitivity analysis was conducted using the leave-one-out method to assess the robustness of the pooled results.

In the network meta-analysis, a network geometry plot was constructed to visualize the comparative relationships among different AE protocols (Chaimani et al., 2013). In the presence of open loops in the network, a consistency model was applied. If closed loops were present, further testing for loop inconsistency was conducted to examine the consistency between direct and indirect comparison results. A *p*-value greater than 0.05 indicated good consistency between direct and indirect evidence, and a consistency model was used; if *p* < 0.05, the sources of inconsistency were further investigated. Subsequently, surface under the cumulative ranking curve (SUCRA) was employed to rank the exercise protocols in order to identify the optimal intervention. Lastly, a comparison-adjusted funnel plot was used to assess potential publication bias and small sample effects (Higgins et al., 2012).

## 3 Results

### 3.1 Literature search and screening

**Figure 1:**
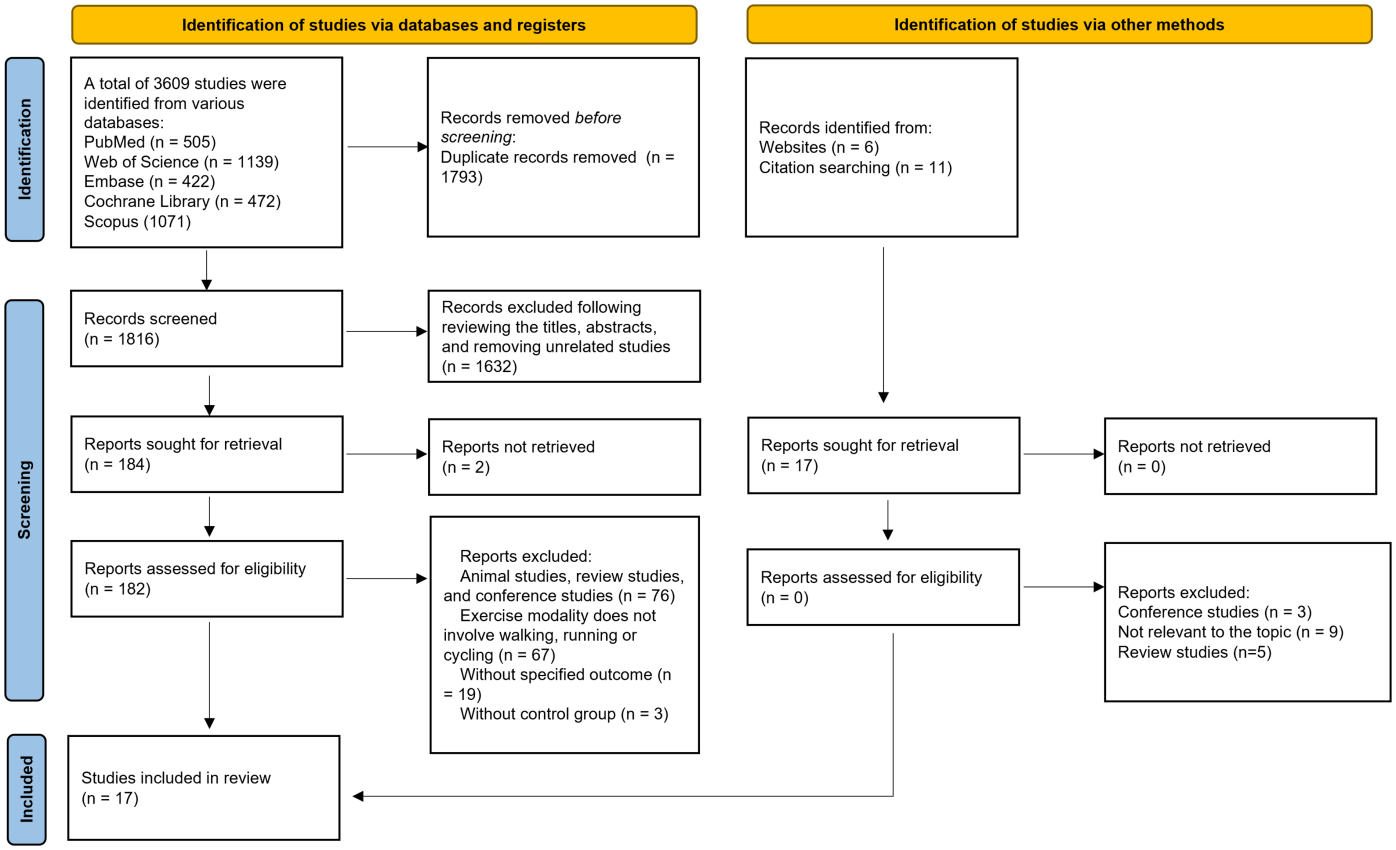
Flowchart of the literature screening process.

The primary flow of literature screening is illustrated in Figure 1. After initial retrieval, removal of duplicates, title and abstract screening, and full-text assessment, a total of 17 studies met the inclusion criteria (Chou et al., 2022; Enette et al., 2020; Erickson et al., 2011; Fungwe et al., 2019; Hsu et al., 2021; Kohanpour et al., 2017; Kovacevic et al., 2020; Li et al., 2021; Lin et al., 2025; Maass et al., 2016; Matura et al., 2017; Osali, 2020; Ploydang et al., 2023; Rackoll et al., 2024; Reed et al., 2022; Segura et al., 2020; Stein et al., 2023).

### 3.2 Characteristics and quality assessment of included studies

**Figure 2:**
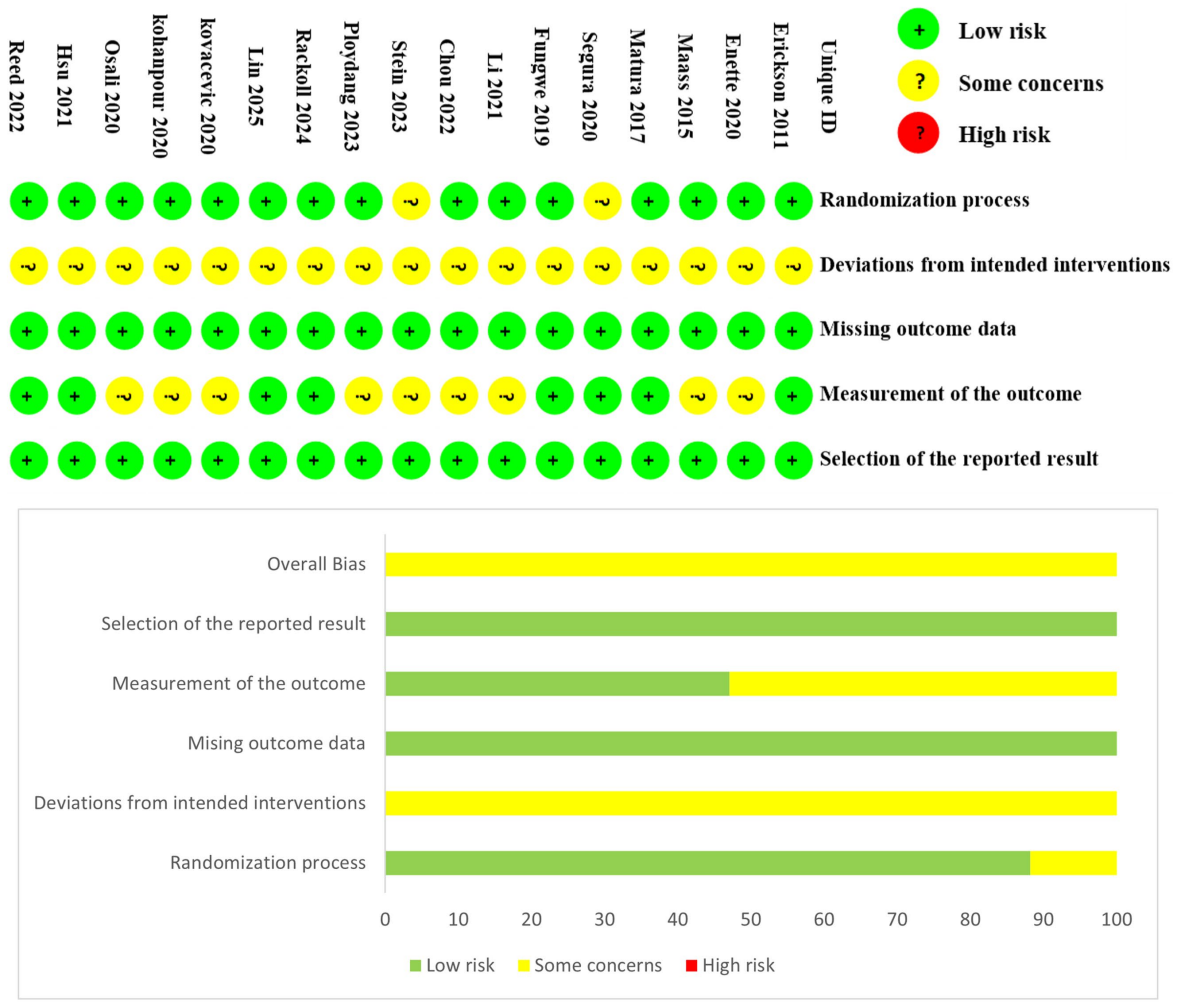
Risk of bias and methodological quality of the included studies.

The 17 included studies involved a total of 900 participants. Among these, 15 studies were incorporated into the traditional meta-analysis, while 2 studies, which involved specific intervention comparisons, were included in the network meta-analysis (Hsu et al., 2021; Reed et al., 2022). Overall, in the included studies, the mean age of participants was 69 years. The exercise interventions involved walking, running, and cycling, with mean session lasting approximately 38 min, performed 2–5 times per week. Mean adherence to the exercise protocols was reported to be 86.4%. Exercise intensity was primarily measured using heart rate or oxygen consumption, ranging from 40 to 95% of heart rate and 60–90% of oxygen uptake. The control groups received interventions such as stretching or relaxation, routine care or health education, or were instructed to maintain their routine. Detailed characteristics of the studies are presented in Table 2. The risk of bias and methodological quality assessments are shown in Figure 2. Notably, due to the inherent nature of exercise interventions, the implementation of double-blind RCTs poses considerable challenges; consequently, most studies were rated as “some concerns” in the second domain of the RoB 2 (Cheng et al., 2025).

**Table 2:** The basic characteristics of included studies.

Participant characteristics							Intervention parameters						Outcome measures
Author/year	Group	Exercise type	Samples	Heath status	Mean ages (year)	BMI (kg/m^2^)	Period	Intensity	Adherence (%)	Frequency	Duration (min)	Rest (min) × Bouts	Measurement
Erickson et al. (2011)	Walking	Supervised aerobic walking	60	Healthy	67.6 ± 5.81	N	1 year	50–75%HRR	79.5 ± 13.7	N	50	N	Serum
	Control	Stretching	60		65.5 ± 5.44				78.6 ± 13.61	N	N		
Enette et al. (2020)	Cycling	Supervised continuous aerobic training	14	Alzheimer’s disease	74 ± 3.75	23 ± 1.25	9 weeks	70%HRmax	87.5	2	30	N	Plasma
	Cycling	Supervised interval aerobic training	17		79 ± 1.75	22 ± 1.00		80%HRmax	100	2	30	4 min × 6	
	Control	Not engage in any exercise training	21		79 ± 2.25	23 ± 1.25		N	100	N	N	N	
Maass et al. (2016)	Walking/Running	Treadmill interval training	21	Healthy	68.4 ± 4.3	N	3 months	65-85%HRmax	100	3	30	2 min	Serum/plasma
	Control	Supervised progressive muscle relaxation/stretching training	19		68.4 ± 4.3			N	100	2	45	N	
Matura et al. (2017)	Cycling	Supervised cycle ergometer training	29	Healthy	73.3 ± 5.5	26.4 ± 4.5	12 weeks	64 ± 9%VO2max	96.7	3	30	N	Serum
	Control	Not to change their habitual physical activity	24		77 ± 8.1	25.8 ± 2.9		N	80	N	N	N	
Segura et al. (2020)	Cycling	Performed high-intensity forced cycling on a stationary tandem bicycle	6	Parkinson’s Disease	57.8	27.5	16 weeks	80%HRmax	N	3	45	N	Plasma
	Control	Non-exercise control	7		56	26.7		N	N	N	N	N	
Fungwe et al. (2019)	Walking/Running	Supervised aerobic exercise	10	Mild Cognitively Impaired	72.1 ± 6.94	27.34 ± 4.36	6 months	70%VO2max	71.4	3	40	N	Serum
	Control	Stretching	7		69.89 ± 7.18	31.64 ± 3.46		N	46.7	N	N	N	
Li et al. (2021)	Cycling	High-intensity interval training	10	Healthy	64.9 ± 3.54	27.8 ± 1.04	12 weeks	90% VO₂max	90.9	3	45	3 min × 4	Serum
	Cycling	Vigorous-intensity continuous training	10		66.4 ± 4.50	27.7 ± 2.84		70% VO₂max	90.9	3	45	N	
	Control	lived normally and did not participate in any training	9		63.9 ± 3.95	27.1 ± 1.50		N	81.8	N	N	N	
Chou et al. (2022)	Walking	Guided Aerobic Walking	30	Hypertensive	69.3 ± 6.5	23.6 ± 2.6	24 weeks	N	90.9	5	30	N	Plasma
	Control	Routine care and a manual on healthy living	28		69.8 ± 7.6	24.9 ± 3.1		N	84.8	N	N	N	
Stein et al. (2023)	Walking/Running	Supervised Treadmill Aerobic Training	18	Alzheimer’s Disease	75.27 ± 6.09	N	12 weeks	75%HRmax	81.8	3	25–40	N	Plasma
	Control	Maintained their routine	16		75.06 ± 6.36	N		N	80	N	N	N	
Ploydang et al. (2023)	Walking	Nordic walking with poles	16	Type 2 Diabetes	68.9 ± 3.7	24.2 ± 3.6	12 weeks	40-60%HRR	94.4	3	60	N	N
	Control	Maintained their routine	17		69.2 ± 5.3	24.8 ± 2.7		N	94.4	N	N	N	
Rackoll et al. (2024)	Walking	Treadmill-based aerobic exercise	60	Stroke	69 ± 12	N	4 weeks	55-65%HRmax	N	~5	21	N	Serum
			45			N		55-65%HRmax		~5	21	N	
	Control	Muscle relaxation	59		70 ± 11	N		N		N	N	N	
			36			N		N		N	N	N	
Lin et al. (2025)	Walking	Supervised muscle stretching and conditioning walking training	18	Mild Cognitive Impairment	85.28 ± 4.65	N	12 weeks	55-65%HRmax	90	3	30	N	Serum
	Control	Routine care and maintenance of daily life activities	18		81.96 ± 6.12	N		N	90	N	N	N	
Kovacevic et al. (2020)	Walking	High-intensity interval training	21	Healthy	72.4 ± 4.4	27 ± 4	12 weeks	90-95%HRmax	75	3	43	3 min × 4	Serum
	Walking	Moderate continuous training	20		72.0 ± 6.2	28 ± 4	12 weeks	70-75%HRmax	74.1	3	52	N	
	Control	Stretching	23		71.5 ± 6.6	30 ± 6	12 weeks	N	82.1	N	N	N	
Kohanpour et al. (2017)	Running	Continuous aerobic running	10	Mild Cognitive Impairment	67.85 ± 3.89	25.69 ± 0.54	12 weeks	75–85%HRR	N	3	26	N	Serum
	Control	Non-exercise control	10					N		N	N	N	
Osali (2020)	Walking/Running	Performed walking or running exercises on a treadmill	11	Metabolic Syndrome	62.3 ± 1.23	29.5 ± 1.2	6 weeks	65–75% HRR	N	N	46–61	5 min × 3	Plasma
	Control	Non-exercise control	11					N		N	N	N	
Hsu et al. (2021)	Cycling	High-intensity interval training on a cycle ergometer	10	Stroke	58.5 ± 20.06	25.5 ± 5.19	12–18 Week	80%VO₂peak	76.9	2–3	30	3 min × 5	Serum
	Cycling	Moderate continuous training on a cycle ergometer	13		53.1 ± 15.91	26.2 ± 5.65		60%VO₂peak	86.7	2–3	30	N	
Reed et al. (2022)	Running/Cycling	Modified Norwegian aerobic HIIT protocol	43	Coronary Artery Disease	61 ± 7	29.0 ± 5.8	12 Weeks	85–95% HRmax	100	2	45	3 min × 4	Plasma
	Walking	Nordic walking with poles	43		61 ± 8	29.3 ± 4.9		Rest Heart + 20–40	100	2	60	N	

Data are presented as mean ± standard deviation; HRR, Heart Rate Reserve; HRmax, Maximum Heart Rate; VO2max, Maximum Oxygen Uptake; VO₂peak, Peak Oxygen Uptake; N, Not Reported.

### 3.3 Meta-analysis

#### 3.3.1 Pooled effect size results

**Figure 3:**
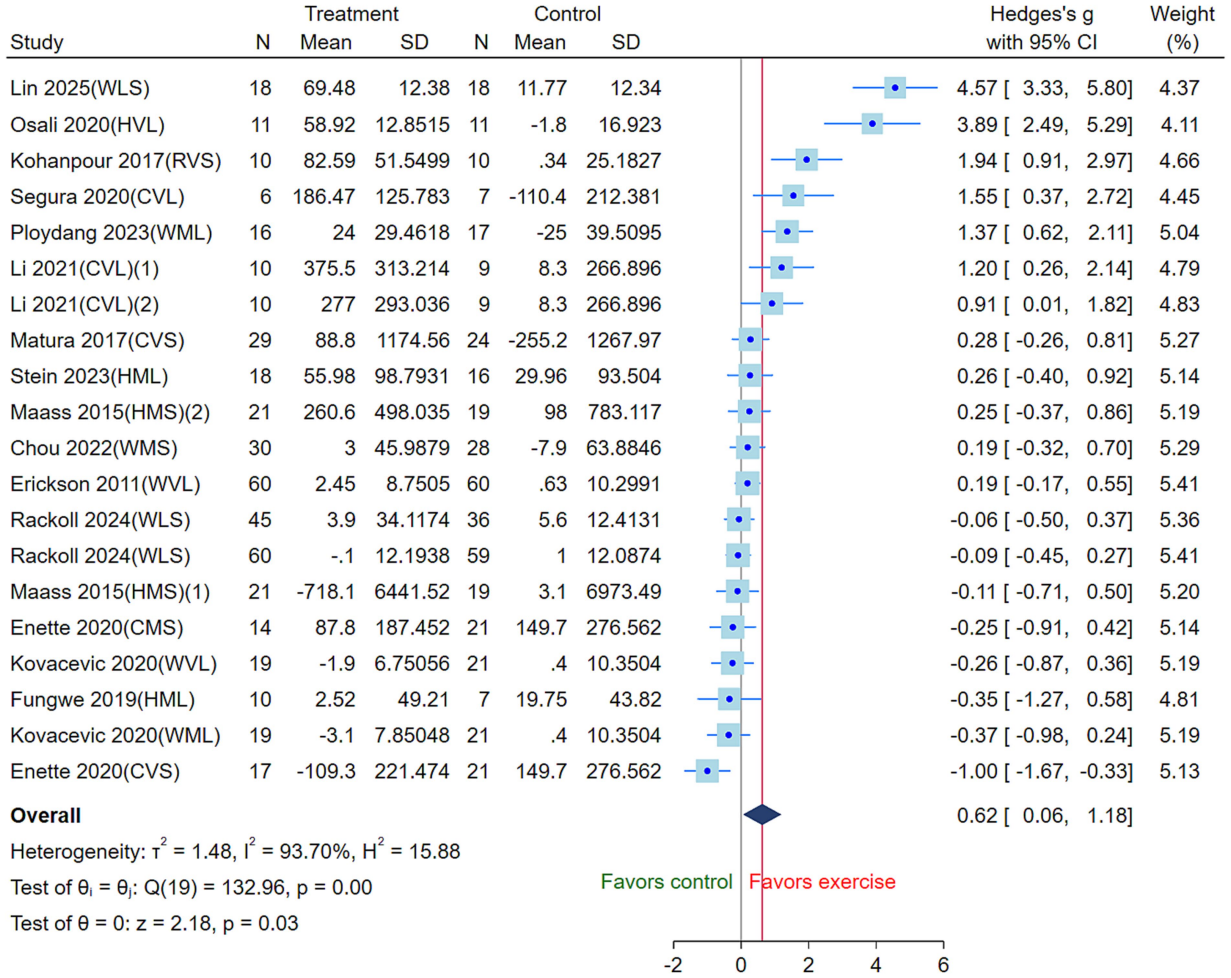
Effects of three aerobic exercise modalities on circulating BDNF levels in the elderly (CVS, high-intensity short-duration cycling; CVL, high-intensity long-duration cycling; CMS, moderate-intensity short-duration cycling; WVL, high-intensity long-duration walking; WMS, moderate-intensity short-duration walking; WML, moderate-intensity long-duration walking; WLS, low-intensity short-duration walking; RVS, high-intensity short-duration running; HVL, high-intensity long-duration mixed exercise; HMS, moderate-intensity short-duration mixed exercise; HML, moderate-intensity long-duration mixed exercise) (Square, the Hedges’s g for study; CI, Confidence Interval; τ^2^, tau-squared; I^2^, I-squared; H^2^, H-squared; Q, Q statistics; z, Z-score).

Interventions were coded according to exercise modality (walking [W], running [R], cycling [C] and Mix [H]), exercise intensity (low [L], moderate [M], high [V]) (Gholami et al., 2025), and intervention duration (greater than 30 min [L] and less than or equal to 30 min [S]) to facilitate network comparison. The heterogeneity test revealed I^2^ = 93.70%, τ^2^ = 1.48, Q(19) = 132.96, *p* < 0.01, indicating significant heterogeneity among the studies. Therefore, a random-effects model was employed to pool the effect sizes. The results showed that the three AE modalities significantly increased circulating BDNF levels compared with the control group (SMD = 0.62, 95% CI: 0.06 to 1.18, *p* = 0.03) (Figure 3).

#### 3.3.2 Subgroup analysis and meta-regression

To further investigate potential sources of heterogeneity and the effects of different variables on BDNF intervention outcomes, subgroup analyses were conducted for categorical variables (Table 3), while meta-regression was performed for continuous variables (Table 4). Regarding intervention modality, running demonstrated a positive effect on BDNF levels in the elderly (Kohanpour et al., 2017), with its 95% CI crossing the null effect line. However, this result was based on a single study and should be interpreted with caution. Other exercise modalities showed positive but non-significant effects (*p* > 0.05), with no significant differences between groups (*p* > 0.05). Subgroup analysis by health status indicated that exercise interventions yielded some beneficial effects on BDNF levels in healthy individuals (Erickson et al., 2011; Kovacevic et al., 2020; Li et al., 2021; Maass et al., 2016; Matura et al., 2017) and those with MCI (SMD > 0) (Fungwe et al., 2019; Kohanpour et al., 2017; Lin et al., 2025), whereas no significant effects were observed in individuals with AD (Enette et al., 2020; Stein et al., 2023) or stroke (Rackoll et al., 2024). Notably, significant differences were found across health status subgroups (*p* < 0.01), suggesting that health status is an important moderator of BDNF intervention effects.

**Table 3:** Subgroup analysis of categorical variables.

Variable	Supgroup	K	Hedges’g	95%CI	P_d_	P_m_
AE modalities	Cycling	6	0.38	[−0.38, 1.13]	0.328	0.11
	Running	1	1.94	[0.91, 2.97]	NA	
	Walking	8	0.63	[−0.42, 1.68]	0.242	
	Mixed	5	0.71	[−0.70, 2.12]	0.324	
Health status	Alzheimer’s disease	3	−0.33	[−1.05, 0.39]	0.373	**0.000**
	Health	8	0.17	[−0.10, 0.43]	0.229	
	Hypertensive	1	0.19	[−0.32. 0.70]	NA	
	Metabolic syndrome	1	3.89	[2.49, 5.29]	NA	
	Mild cognitive impairment	3	2.03	[−0.75, 4.80]	0.152	
	Parkinson’s disease	1	1.55	[0.37, 2.72]	NA	
	Stroke	2	−0.08	[−0.35, 0.20]	0.574	
	Type 2 diabetes	1	1.37	[0.62, 2.11]	NA	
Gender	Female	3	1.26	[−1.16, 3.68]	0.308	0.79
	Male	2	0.87	[−1.12, 2.85]	0.393	
	Male and female	15	0.49	[−0.12, 1.09]	0.115	
Intensity	Light	3	1.42	[−1.56, 4.40]	0.351	0.22
	Moderate	8	0.12	[−0.24, 0.48]	0.5	
	High	9	0.88	[0.00, 1.75]	**0.05**	
Intermittent	Not have	16	0.57	[0.02, 1.11]	**0.041**	0.76
	Have	4	0.89	[−1.17, 2.95]	0.396	
Measurement	Plasma	7	0.61	[−0.48, 1.69]	0.273	0.33
	Serum	12	0.59	[−0.14, 1.32]	0.116	
	Not report	1	1.37	[0.62, 2.11]	NA	

K, Number of studies; P_d_, Significance level within a single subgroup; P_m_, Significance level between subgroups. Bold values indicate *p* ≤ 0.05.

**Table 4:** Meta-regression of continuous variables.

Predictor	Coefficient (β)	Std. Error	95% CI	z	*p*-value
Age	−0.0269	0.0519	[−0.1287, 0.0749]	−0.52	0.604
BMI	0.1541	0.1456	[−0.1313, 0.4394]	1.06	0.29
Frequency	−0.0587	0.3175	[−0.6809, 0.5636]	−0.18	0.853
Duration	0.0266	0.0259	[−0.0243, 0.0776]	1.02	0.306
Period	−0.0142	0.0283	[−0.0697, 0.0413]	−0.5	0.616

Std. Error, Standard Error.

Subgroup analysis by sex revealed no significant differences between males and females (*p* > 0.05). In terms of exercise intensity, the high-intensity exercise group showed a borderline positive effect (*p* = 0.05), while the low and moderate-intensity groups did not reach statistical significance (*p* > 0.05), and the differences between these groups were not significant (*p* > 0.05). Additionally, the non-intermittent exercise group exhibited a significant intervention effect (*p* < 0.05), while the intermittent exercise group showed no significant difference (*p* > 0.05), with no significant differences between the groups (*p* > 0.05). Finally, no statistical significance was found regardless of whether the measurement site was plasma or serum, and the differences between groups were not significant (*p* > 0.05).

**Figure 4:**
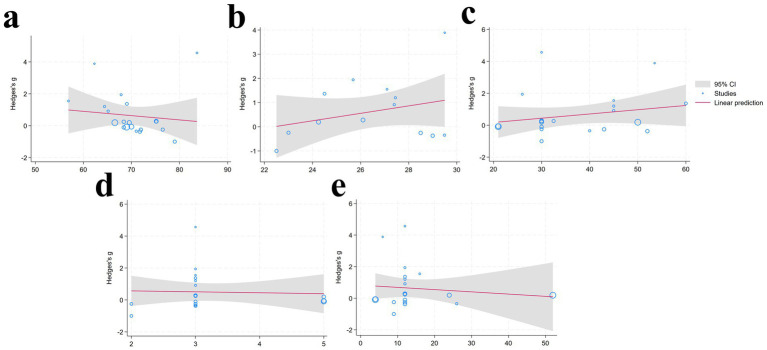
Meta-regression analysis results [**(a)**: Age; **(b)** BMI; **(c)** duration; **(d)** frequency; **(e)** period] (95% CI, Gray shaded region around the regression line; Studies, Blue circular; Linear prediction, Red solid line depicting the fitted linear regression model).

The results of the meta-regression indicate that variables including age, BMI, duration (min), frequency, and period (weeks) did not have a significant effect on the intervention outcomes (*p* > 0.05), indicating that these variables are unlikely to be primary moderators of the intervention effects (Figure 4) (Table 4).

#### 3.3.3 Publication bias and sensitivity analysis

**Figure 5:**
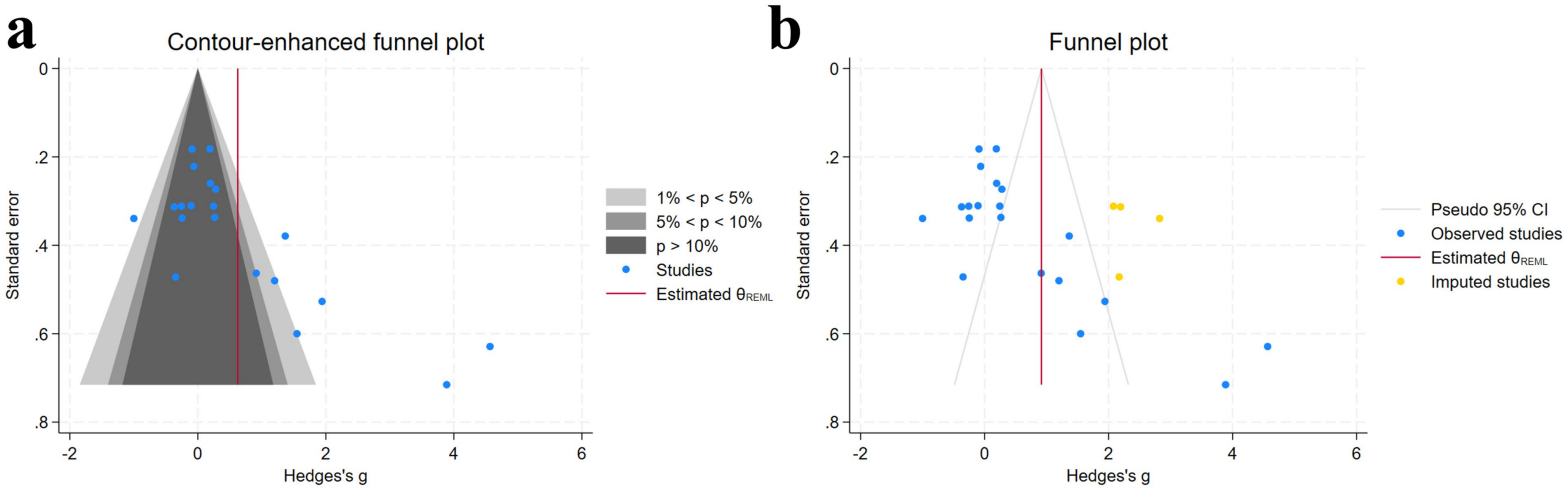
Funnel plot and trim-and-fill method [**(a)** funnel plot; **(b)** trim-and-fill method] [Gray regions, Statistical significance contours; Blue dots, Individual studies included in the meta-analysis; Red line, Estimated overall effect size (*θREML*); Yellow dots, Imputed studies (data imputed to address missing studies or publication bias)].

Visual inspection of the funnel plot suggested the presence of potential publication bias, which was supported by the Egger test (*p* < 0.05) (Supplementary material). To address this, we used the trim-and-fill method to impute missing studies. After adding three missing studies, the Hedges’ g and its 95% CI increased from 0.624 (0.063 to 1.184) to 0.917 (0.373 to 1.460), and the results remained significant, indicating that the potentially missing studies did not significantly affect the overall effect (Figure 5). Subsequently, a leave-one-out meta-analysis was performed by removing each study (Supplementary material). The results showed that regardless of which study was excluded, the pooled effect size remained within the 95% CI, confirming the robustness and reliability of the findings.

### 3.4 Network meta-analysis

#### 3.4.1 Network evidence diagram

**Figure 6:**
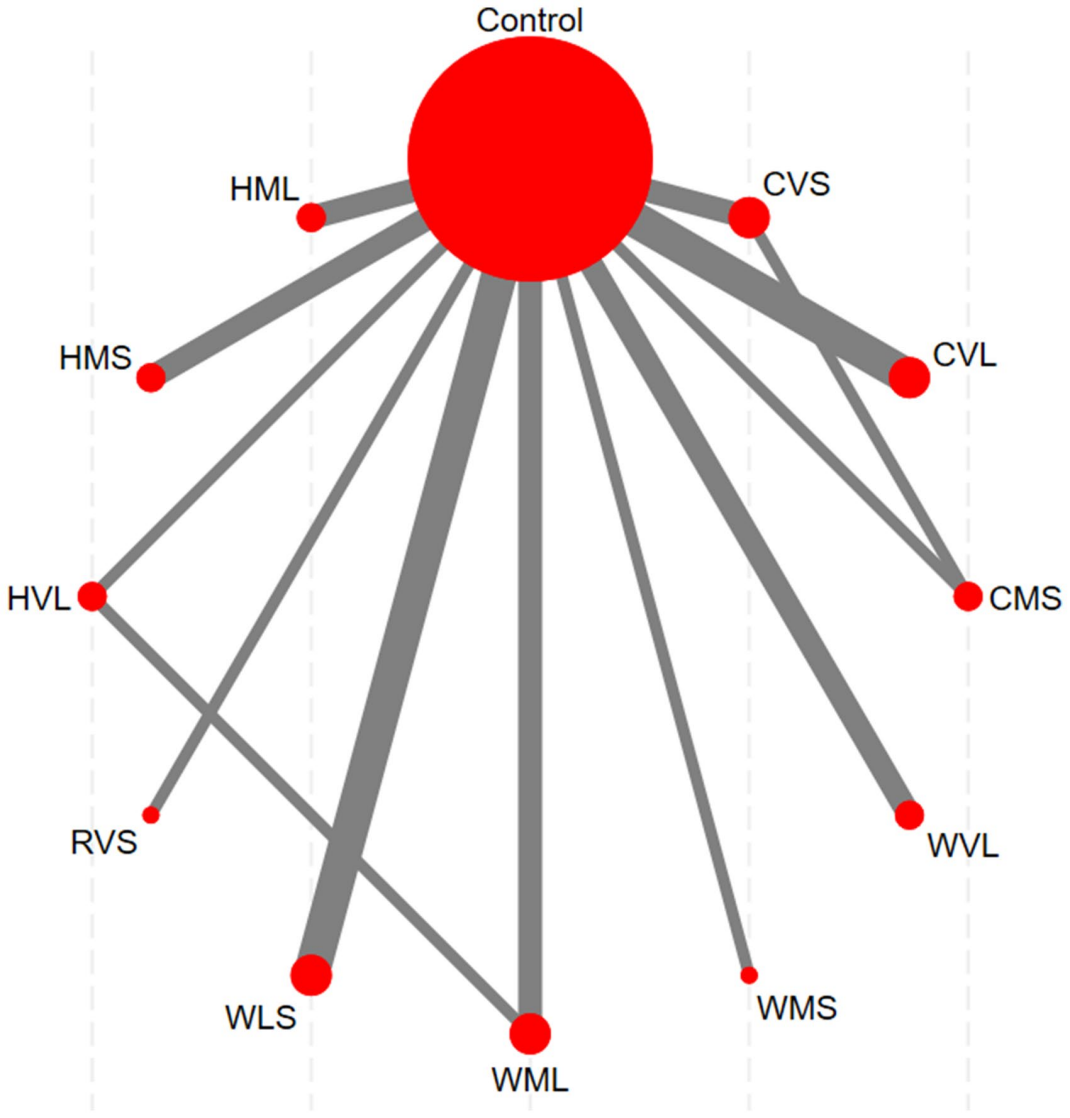
Network evidence diagram (Red nodes: represent individual interventions; Gray lines: indicate direct comparisons between two interventions).

The network evidence diagram is presented in Figure 6. Each node in the diagram represents an intervention, with the node size proportional to the sample size involved in the corresponding intervention. The lines connecting nodes indicate the existence of direct comparisons between two interventions, and the thickness of each line corresponds to the number of such direct comparisons. The network evidence diagram reveals direct comparisons between CVS and CMS, as well as between HVL and WML, whereas other intervention pairs lack direct comparisons and therefore require indirect comparison through network meta-analysis.

#### 3.4.2 Pairwise comparisons between protocols

**Figure 7:**
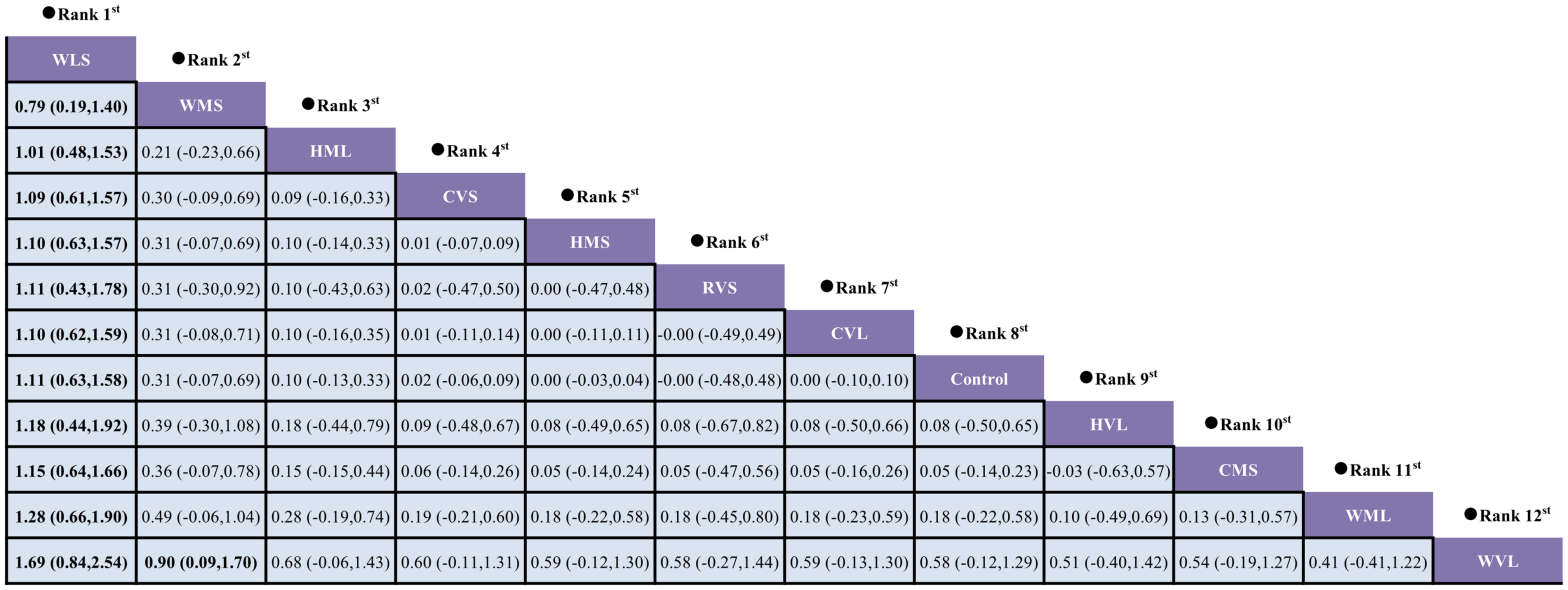
Pairwise comparisons between exercise interventions.

**Figure 8:**
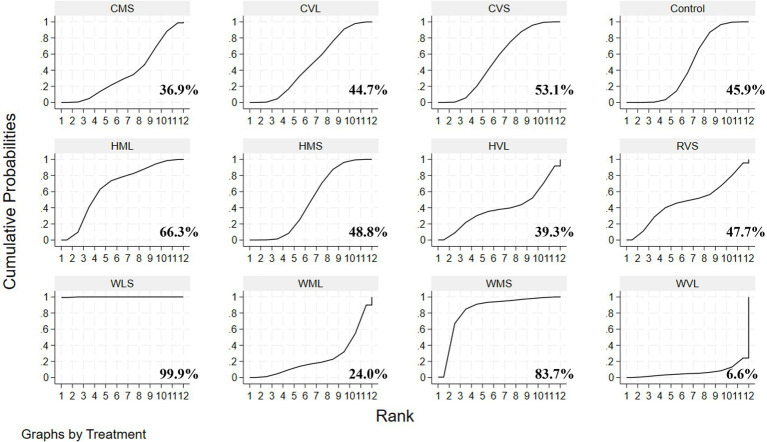
SUCRA rankings of various exercise protocols.

**Figure 9:**
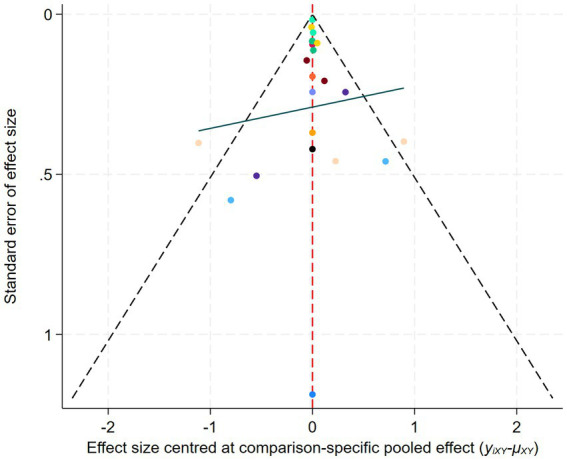
Comparison-Adjusted Funnel Plot of Network Meta-Analysis.

A loop inconsistency test was conducted to examine the consistency between direct and indirect evidence. The results showed good consistency among comparisons (*p* > 0.05); therefore, a consistency model was used for the network meta-analysis. Results indicated that protocols involving WLS was superior to other AE protocols; additionally, protocols involving WMS also outperformed WVL (SMD = 0.90, 95% CI: 0.09 to 1.70). These comparisons reached statistical significance (*p* < 0.05) (Figure 7). Based on SUCRA rankings, protocols involving WLS (99.9%) and WMS (83.7%) were the top-ranked interventions (Figure 8). Finally, visual inspection of the funnel plot showed no obvious publication bias (Figure 9).

## 4 Discussion

This study systematically evaluated the effects of walking, running, and cycling on circulating BDNF levels in elderly. The meta-analysis results indicated that three AE modalities significantly improved circulating BDNF levels in elderly. Although our review did not directly assess cognition, neuroplasticity, or neurodegeneration, existing evidence suggests that AE may have potential benefits for these functions through BDNF and could help slow age-related decline in brain function (Hayes et al., 2014). It is noteworthy that the degree of improvement in BDNF levels was significantly influenced by the participants’ health status, suggesting that health status may be an important modulating factor in the impact of AE on circulating BDNF. Specifically, the interventions had positive effects in healthy individuals and those with MCI, whereas the effects were less evident in individuals with AD or stroke. Furthermore, training variables such as the modality of intervention, exercise intensity, and interval may also serve as potential influencing factors. To compare the relative advantages of different AE protocols in improving BDNF levels, a network meta-analysis was further conducted. Pairwise comparisons and SUCRA results revealed that exercise protocols involving low- to moderate-intensity walking (e.g., WLS vs. WMS) may exert the most favorable effect on circulating BDNF. These findings have important practical implications, suggesting that when developing individualized exercise prescriptions to improve BDNF in the elderly, priority should be given to sustainable walking interventions of appropriate intensity.

A large body of research has demonstrated that AE is an effective non-pharmacological intervention for improving cognitive impairment in older adults, and it is widely used in the prevention and treatment of neurological diseases such as MCI, AD, and PD (Baker et al., 2010; Segura et al., 2020; Yang et al., 2025). In fact, these benefits are largely attributed to the ability of AE to promote neuroplasticity. Neuroplasticity refers to the brain’s capacity to change and adapt based on experiences and environmental factors. Specifically, it can modify its structure and form new neural networks, with BDNF expression being a key regulatory mechanism (Ben Ezzdine et al., 2025; Khalil, 2025). Research has shown that AE can enhance the release of BDNF, thereby improving dendritic spine integrity and activating various signaling pathways associated with neuroplasticity. Specifically, BDNF promotes dendritic spine formation by activating TrkB receptors, which trigger a series of signaling pathways such as PI3K/Akt, RAS/ERK, and PLCγ1/PKC (Alonso et al., 2004).

The present study further demonstrates that the three AE modalities elicit varying degrees of improvement in circulating BDNF levels across different health statuses. Specifically, positive intervention effects (SMD > 0) are observed in healthy individuals and those with MCI, hypertension, metabolic syndrome (MetS), PD, and T2D, whereas in patients with AD and stroke, AE does not have a positive effect on BDNF levels (SMD < 0). Previous studies have demonstrated that AD patients exhibit extracellular deposition of amyloid-*β* plaques and intracellular hyperphosphorylation of tau protein. These pathological features disrupt axonal transport of BDNF, reduce its synaptic availability, and impair TrkB receptor-mediated signaling pathways, potentially negatively impacting circulating BDNF levels (Lima Giacobbo et al., 2019). Additionally, platelets serve as a major reservoir of BDNF (Boukhatem et al., 2024), and skeletal muscle can secrete BDNF during physical activity (Schnyder and Handschin, 2015). In AD patients, reduced physical activity and muscle atrophy may lead to decreased release of BDNF from both platelets and skeletal muscle into the circulation, further contributing to lower peripheral BDNF levels (Knaepen et al., 2010). Similar to AD, patients with MCI also exhibit significantly lower circulating BDNF levels compared to healthy individuals, suggesting that early stages of neurodegenerative disease may negatively affect BDNF expression and function (Miranda et al., 2019). In PD, reduced mRNA and protein expression of BDNF in the substantia nigra pars compacta has also been observed (Howells et al., 2000), further supporting the notion that neurodegenerative disorders adversely impact neural structure and function by impairing BDNF signaling.

Beyond these conditions, evidence has shown that individuals with coronary heart disease, T2D, MetS, or sedentary lifestyles also exhibit a generally reduced peripheral BDNF levels (Huang et al., 2014), which may be closely linked to their lower cardiorespiratory fitness and adverse metabolic status. These conditions are frequently characterized by chronic low-grade inflammation, insulin resistance, endothelial dysfunction, and insufficient physical activity (Booth et al., 2012). Collectively, these pathophysiological alterations are likely to impair the synthesis and release of BDNF, thereby contributing to diminished peripheral BDNF concentrations (Arentoft et al., 2009; Júdice et al., 2021; Motamedi et al., 2017; Shobeiri et al., 2023). Lower circulating BDNF concentrations are not only associated with an elevated risk of cardiovascular and cerebrovascular diseases but may also be linked to impaired cognitive recovery (Pikula et al., 2013). Notably, our subgroup analysis revealed that the effects of exercise interventions on BDNF recovery were limited in individuals with AD and stroke, possibly due to irreversible pathological damage in the brain (Ma et al., 2025; Mohd Khairi et al., 2024). It should be noted that the number of studies included in the subgroup analyses for hypertension, AD, MetS, PD, and T2D was relatively small. Therefore, these findings should be interpreted with caution.

Subsequently, a network meta-analysis was conducted to perform pairwise comparisons and SUCRA ranking, revealing that protocols involving walking at low to moderate intensity, with a duration not exceeding 30 min per session (e.g., WLS and WMS), was the most effective for enhancing circulating BDNF levels in older adults. Notably, an intriguing observation emerged during the analysis: the overall ranking results from the network meta-analysis were inconsistent with those obtained from traditional subgroup analyses. Specifically, in the subgroup analysis, higher exercise intensity and continuous (non-intermittent) exercise protocols appeared to positively influence circulating BDNF levels in older adults. However, when exercise modality, intensity, and duration were considered collectively in the network meta-analysis, walking at low to moderate intensity with short-duration interventions emerged as the optimal combination. Moreover, as only two additional studies were included in the network meta-analysis, this may have contributed to the discrepancy between its results and those of the subgroup analysis. It is noteworthy that, as the included studies did not report total exercise volume, the results for sessions lasting less than 30 min should be interpreted with caution.

Another important factor to consider is exercise intensity. According to the classification of Gholami et al. (2025) exercise intensity, Erickson et al. (2011) reported that although 1 year of high-intensity aerobic walking produced some improvements in hippocampal volume, changes in serum BDNF levels did not differ significantly. Furthermore, Kovacevic et al. (2020) investigated the effects of exercise intensity on BDNF and found that after 12 weeks of AE intervention, there were no significant differences in BDNF levels between the HIIT, MICT, and Control groups. In contrast, Ploydang et al. (2023) found that 12 weeks of moderate-intensity aquatic Nordic walking significantly increased serum BDNF. These findings are consistent with our results, suggesting that protocols involving moderate-intensity AE (e.g., aquatic Nordic walking) may be more favorable for enhancing BDNF. The underlying mechanisms may include the following. First, high-intensity AE substantially elevates stress hormones, such as cortisol, which may suppress BDNF expression and offset some positive effects of exercise (Clos et al., 2021; Jeon and Ha, 2015). Second, lactate can stimulate BDNF production via the PGC-1α/FNDC5 pathway; however, excessive lactate accumulation may lead to blood acidification and metabolic stress, thereby limiting circulating BDNF levels (Brooks et al., 2023; Müller et al., 2020).

Exercise duration is another critical factor. Current evidence indicates that even 30-min AE sessions can effectively increase circulating BDNF levels in older adults. For example, Lin et al. (2025) reported that 12 weeks of walking training (55–65% HRmax) significantly elevated serum BDNF. Therefore, we speculate that exercise sessions limited to 30 min may improve BDNF levels through moderate neural stimulation, limited stress hormone release, and optimized lactate signaling, thereby promoting neuroplasticity. However, this interpretation should be made with caution given the high heterogeneity and variability across study samples. Regarding whether AE should be performed with intervals, we did not consider this a primary dimension for categorizing interventions for two main reasons. First, although continuous exercise showed a slight advantage over intermittent exercise in subgroup analyses of BDNF enhancement, inter-group differences were not statistically significant. Second, given that older adults are more susceptible to exercise-induced fatigue and intolerance due to physiological decline, the use of intervals should be flexibly adjusted based on individual responses (Chmelo et al., 2015). These findings indicate that, for older adults, enhancing BDNF levels requires a multifactorial approach, rather than focusing on any single exercise parameter. Future research should further explore the combined effects of exercise intensity, duration, and intervention period to optimize exercise prescriptions and maximize improvements in BDNF levels and cognitive function in older adults.

This study has several limitations that warrant attention. First, although strictly RCTs were included, the inherent characteristics of exercise interventions make it difficult to conduct truly double-blind RCTs in practice. Nevertheless, awareness of group allocation is unlikely to directly affect objective biological outcomes such as blood BDNF levels. Second, the lack of significant subgroup differences for variables such as age, BMI, duration, frequency, or intervention period does not necessarily indicate that these factors have no effect. Rather, this may be due to the narrow ranges represented in the included studies or methodological inconsistencies. Future studies are needed to further clarify the influence of these factors. Moreover, as only two additional studies were included in the network meta-analysis, this may have contributed to the discrepancy between its results and those of the subgroup analysis, representing an additional limitation of the study. Additionally, considerable heterogeneity was observed across studies, which may be related to baseline activity levels, BDNF assay methodology, timing of blood sampling, and adherence rates. For certain outcomes, only a limited number of trials were included in the subgroups, potentially restricting the robustness and generalizability of our findings. Moreover, this study focused on walking, running, and cycling to more clearly assess their effects on BDNF. To reduce heterogeneity and improve comparability, we excluded activities like dancing and Tai Chi, which are more variable and harder to standardize. While this strengthened the internal validity of our analysis, it may limit the generalizability of the findings. Finally, most studies currently use plasma or serum BDNF levels as assessment indicators. While previous studies have indicated that peripheral BDNF can partially reflect brain BDNF levels, this indirect measurement method still has limitations and may be influenced by external factors such as sample processing methods. Future research should explore more sensitive and specific measurement techniques for directly assessing brain BDNF levels to enhance the reliability and interpretability of this biomarker.

## 5 Conclusion

Walking, running, and cycling are effective for improving circulating BDNF levels in older adults; however, the magnitude of improvement depends on participants’ health status and specific exercise prescription. Interventions involving walking at low to moderate intensity demonstrated favorable efficacy. This effect may be more favorable in healthy individuals and those with mild cognitive impairment. Future studies should further investigate the influence of total exercise volume on outcomes and adopt more rigorous and standardized protocols to facilitate the development of standardized exercise strategies, thereby improving comparability and reducing heterogeneity in future analyses.

## Data Availability

The original contributions presented in the study are included in the article/Supplementary material, further inquiries can be directed to the corresponding author.
