# Supplementary material for: Effects of three aerobic exercise modalities (walking, running, and cycling) on circulating brain-derived neurotrophic factor in older adults: a systematic review and meta-analysis
Source: Front Aging Neurosci. 2025 Sep 25;17:1673786. doi: 10.3389/fnagi.2025.1673786 (PMC12507801; doi:10.3389/fnagi.2025.1673786)
Supplement: Supplementary file 1 [file Supplementary_file_1.docx]

Supplementary Material

# Supplementary Table

**Supplementary Table 1:** **Search terms used in this study**

**Pubmed (505)**

| #1 | (((((((((((((((((((((("Exercise"[Mesh]) OR (Exercises[Title/Abstract])) OR (Exercise, Physical[Title/Abstract])) OR (Exercises, Physical[Title/Abstract])) OR (Physical Exercise[Title/Abstract])) OR (Physical Exercises[Title/Abstract])) OR (Exercise, Aerobic[Title/Abstract])) OR (Aerobic Exercise[Title/Abstract])) OR (Aerobic Exercises[Title/Abstract])) OR (Aerobic training[Title/Abstract])) OR (Exercises, Aerobic[Title/Abstract])) OR (Exercise Training[Title/Abstract])) OR (Exercise Trainings[Title/Abstract])) OR (Training, Exercise[Title/Abstract])) OR (Trainings, Exercise[Title/Abstract])) OR (Physical Activity[Title/Abstract])) OR (Activities, Physical[Title/Abstract])) OR (Activity, Physical[Title/Abstract])) OR (Physical Activities[Title/Abstract])) OR (Exercise[Title/Abstract])) OR ((("Running"[Mesh]) OR (running[Title/Abstract])) OR (Jogging[Title/Abstract]))) OR ((("Walking"[Mesh]) OR (Nordic Walking[Title/Abstract])) OR (Walking[Title/Abstract]))) OR ((("Bicycling"[Mesh]) OR (Cycling[Title/Abstract])) OR (Bicycling[Title/Abstract])) |
| --- | --- |
| #2 | ((((((((("Aged"[Mesh]) OR (Aged[Title/Abstract])) OR (Elders[Title/Abstract])) OR (Elder[Title/Abstract])) OR (Older Adults[Title/Abstract])) OR (Adult, Older[Title/Abstract])) OR (Adults, Older[Title/Abstract])) OR (Older Adult[Title/Abstract])) OR (Elderly[Title/Abstract])) |
| #3 | ((((("Brain-Derived Neurotrophic Factor"[Mesh]) OR (Brain-Derived Neurotrophic Factor[Title/Abstract])) OR (Brain Derived Neurotrophic Factor[Title/Abstract])) OR (Factor, Brain-Derived Neurotrophic[Title/Abstract])) OR (Neurotrophic Factor, Brain-Derived[Title/Abstract])) OR (BDNF[Title/Abstract]) |
| #4 | #1 AND #2 AND #3 |

**Web of Science (1139)**

| #1 | ((((((((((((TS=(Exercise)) OR TS=(Exercises)) OR TS=(Physical Exercise)) OR TS=(Aerobic Exercise)) OR TS=(Aerobic training)) OR TS=(Exercise Training)) OR TS=(Physical Activity)) OR TS=(Running)) OR TS=(Jogging)) OR TS=(Walking)) OR TS=(Nordic Walking)) OR TS=(Bicycling)) OR TS=(Cycling) |
| --- | --- |
| #2 | (((TS=(Aged)) OR TS=(Elder)) OR TS=(Older Adult)) OR TS=(Elderly) |
| #3 | ((TS=(Brain-Derived Neurotrophic Factor)) OR TS=(Brain Derived Neurotrophic Factor)) OR TS=(BDNF) |
| #4 | #1 AND #2 AND #3 |

**Cochrane Library (472)**

| #1 | exercise |
| --- | --- |
| #2 | Physical Exercise |
| #3 | physical activity |
| #4 | Aerobic Exercise |
| #5 | Exercise Training |
| #6 | Running |
| #7 | Cycling |
| #8 | Bicycling |
| #9 | Nordic Walking |
| #10 | Walking |
| #11 | Jogging |
| #12 | Aerobic training |
| #13 | #1 or #2 or #3 or #4 or #5 or #6 or #7 or #8 or #9 or #10 or #11 or #12 |
| #14 | Brain Derived Neurotrophic Factor |
| #15 | Brain-Derived Neurotrophic Factor |
| #16 | BDNF |
| #17 | Aged |
| #18 | Elder |
| #19 | Older Adult |
| #20 | Elderly |
| #21 | #14 or #15 or #16 |
| #22 | #17 or #18 or #19 or #20 |
| #23 | #13 and #21 and #22 |

**Embase (422)**

| #1 | exercise:ab,ti OR 'physical exercise':ab,ti OR 'aerobic exercise':ab,ti OR 'aerobic training':ab,ti OR 'exercise training':ab,ti OR 'physical activity':ab,ti OR running:ab,ti OR jogging:ab,ti OR walking:ab,ti OR 'nordic walking':ab,ti OR bicycling:ab,ti OR cycling:ab,ti |
| --- | --- |
| #2 | aged:ab,ti OR elder:ab,ti OR 'older adult':ab,ti OR elderly:ab,ti |
| #3 | 'brain-derived neurotrophic factor':ab,ti OR 'brain derived neurotrophic factor':ab,ti OR bdnf:ab,ti |
| #4 | #1 AND #2 AND #3 |

**Scopus (1071)**

| #1 | ( TITLE-ABS-KEY ( exercise ) OR TITLE-ABS-KEY ( physical AND exercise ) OR TITLE-ABS-KEY ( physical AND activity ) OR TITLE-ABS-KEY ( aerobic AND exercise ) OR TITLE-ABS-KEY ( exercise AND training ) OR TITLE-ABS-KEY ( running ) OR TITLE-ABS-KEY ( cycling ) OR TITLE-ABS-KEY ( bicycling ) OR TITLE-ABS-KEY ( nordic AND walking ) OR TITLE-ABS-KEY ( walking ) OR TITLE-ABS-KEY ( jogging ) OR TITLE-ABS-KEY ( aerobic AND training ) ) |
| --- | --- |
| #2 | ( TITLE-ABS-KEY ( elderly ) OR TITLE-ABS-KEY ( older AND adult ) OR TITLE-ABS-KEY ( elder ) OR TITLE-ABS-KEY ( aged ) ) |
| #3 | ( TITLE-ABS-KEY ( brain-derived AND neurotrophic AND factor ) OR TITLE-ABS-KEY ( brain AND derived AND neurotrophic AND factor ) OR TITLE-ABS-KEY ( bdnf ) ) |
| #4 | #1 AND #2 AND #3 |

# Supplementary Figures

## Supplementary Figures 1: Sensitivity analysis

**
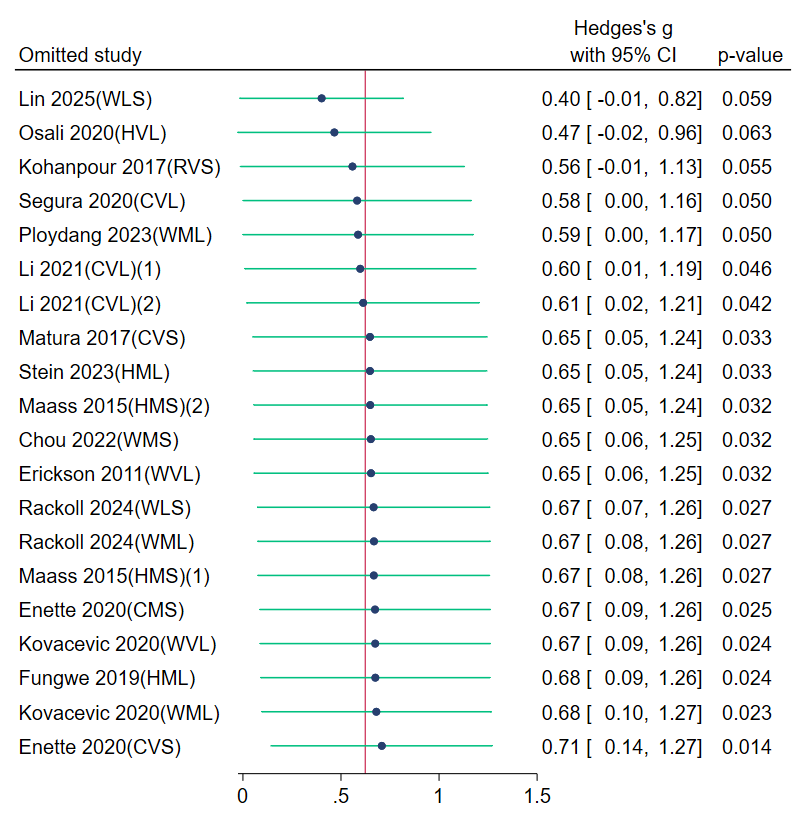
**

## Supplementary Figures 2: Egger Test

**
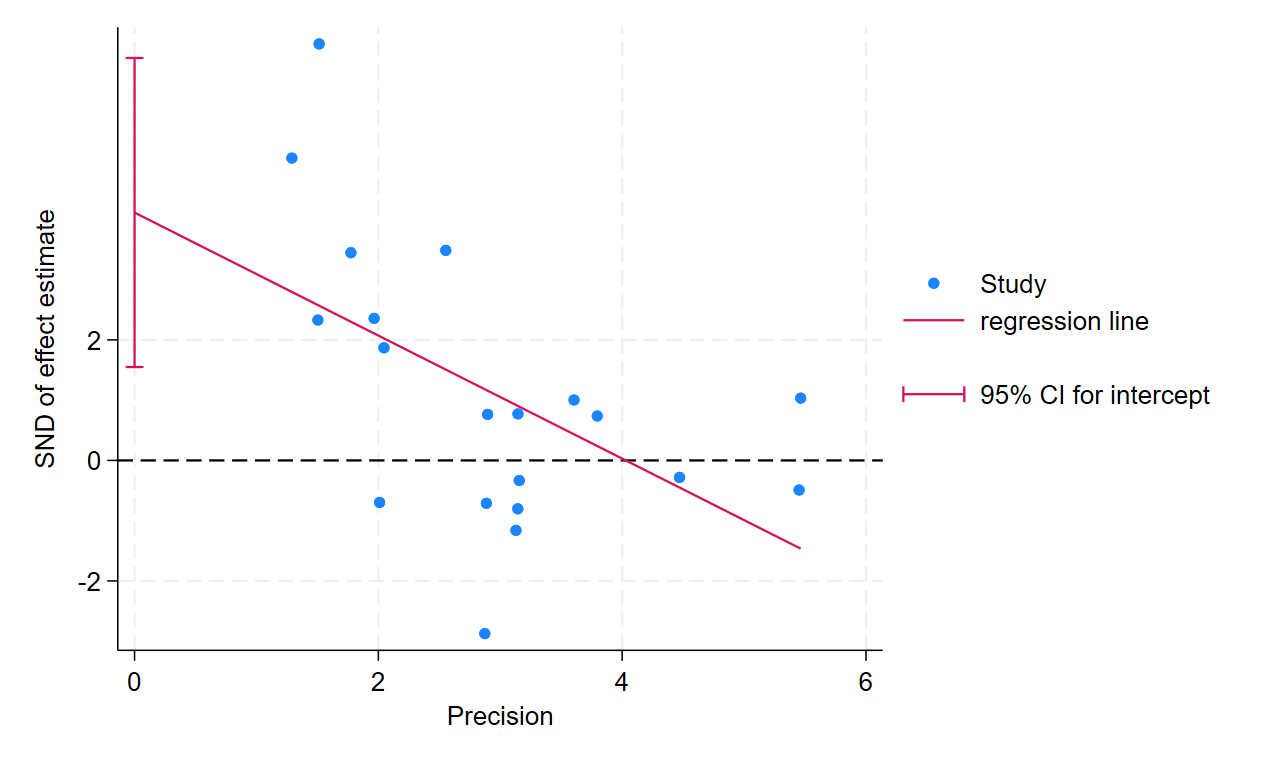
**
